# A Relation Routing Scheme for Distributed Semantic Media Query

**DOI:** 10.1155/2013/915963

**Published:** 2013-11-11

**Authors:** Zhuhua Liao, Guoqiang Zhang, Aiping Yi, Guoqing Zhang, Wei Liang

**Affiliations:** ^1^Key Laboratory of Knowledge Processing and Networked Manufacturing, University of Hunan Province, Xiangtan 411201, China; ^2^School of Computer Science and Technology, Nanjing Normal University, Nanjing 210023, China; ^3^Hunan University of Science and Technology, Xiangtan 411201, China; ^4^Institute of Computing Technology, Chinese Academy of Sciences, Beijing 100190, China

## Abstract

Performing complex semantic queries over large-scale distributed media contents is a challenging task for rich media applications. The dynamics and openness of data sources make it uneasy to realize a query scheme that simultaneously achieves precision, scalability, and reliability. In this paper, a novel *relation routing scheme (RRS)* is proposed by renovating the routing model of Content Centric Network (CCN) for directly querying large-scale semantic media content. By using proper query model and routing mechanism, semantic queries with complex relation constrains from users can be guided towards potential media sources through *semantic guider nodes*. The scattered and fragmented query results can be integrated on their way back for semantic needs or to avoid duplication. Several new techniques, such as semantic-based naming, incomplete response avoidance, timeout checking, and semantic integration, are developed in this paper to improve the accuracy, efficiency, and practicality of the proposed approach. Both analytical and experimental results show that the proposed scheme is a promising and effective solution for complex semantic queries and integration over large-scale networks.

## 1. Introduction

Over years, many research efforts have been devoted to large-scale data retrieving in dynamic self-organized network environments, for example, WWW and various forms of peer-to-peer networks. Database and multimedia technologies provide the means to connect and interact with rich elements of static data, which stimulates media artifacts generation in various kinds of networks. However, since the state of nodes may change at any time, data may be moved from one place to another, and links between data may be broken; many new characteristics of the rich media emerge in these networks. For example, many semantically interrelated data are located on different nodes, numerous distributed data fragments are about the same scenario, and many *knowledge islands* are emerging. These issues pose great challenges to realize efficient data query and integration schemes that support complex semantics. In addition to supporting queries with complex semantics, it is also of equal importance to improve the precision, resilience, and scalability of the data query schemes.

In traditional web search engine based solutions [1, 2], distributed media contents are crawled from the Internet to one or a few sites, and then, a central, big index structure is built for centralized querying after data is extracted and sorted. Although centralized searching can support complex semantic query, it is, however, hard to meet the dynamism of the network itself and the speed of network information updating.

A number of schemata have been proposed to timely search the fully decentralized network data. However, these schemata fail to simultaneously meet the requirements of efficient routing and the ability to express complex semantic queries. According to whether index technique is used, these solutions can be classified into three categories [3]: mechanisms without an index, mechanisms with dedicated index nodes, and mechanisms with indices at each node. According to the semantic capability, these solutions can also be classified into three categories: document-oriented data retrieval, topic searching, and structural or semantic query. Unfortunately, all these solutions that achieve efficient routing only support keywords or topic-based queries; they are not capable of expressing complex structural or semantic queries.

However, in reality, the ability to support distributed semantic query over large-scale networks will enable two promising features: (1) filtering query results by imposing constraints over the content to be searched. In keywords based search, many irrelevant or duplicated results may be returned, whereas with semantic query and data integration, irrelevant results will be pruned at data sources, and duplicated results will be suppressed at intermediate routers; (2) retrieving relevant data by appropriately specifying relation constraints. Relation constraints allow users to retrieve relevant data (e.g., fragmented data) in one query, rather than issuing multiple queries for different data or data parts.

This paper is concerned with the elementary theory and implementation of relation routing for semantic queries over large-scale networks, which can support complex queries over large-scale semantic media that vary with location and semantic schema. The ability of querying and integrating decentralized media over large-scale networks is of great benefit to individual learning, customized media authoring, and so forth. In our schema, applications can complete various semantic queries and sequential integrations over large-scale semantic media without having to build a global semantic view of the media at each node or at a central node in advance. The contributions of this paper are as follows.

- We presented the whole framework of relation routing scheme (RRS) that supports complex semantic query makeup, query routing, and result integration.
- We addressed several fundamental issues that are specific to RRS: semantic-based naming, the incomplete response problem arising from the 1 : *n* relationship between query and potential results, and the relation integration for semantic or performance considerations.
- We provided a prototype implementation of RRS by extending the CCN protocol. We inherited several building blocks or designing principles from CCN, such as the guided information flooding routing model and soft-state design principle, but meanwhile, we also made several extensions to CCN, such as the introduction of Response Relation Table to tackle the incomplete response problem.

The rest of the paper is organized as follows.  Section 2 describes related work.  Section 3 details how to extend the forwarding engine of CCN to realize our relation routing scheme.  Section 4 discusses how to perform relation integration.  Section 5 presents both analytical and experimental performance evaluation of RRS. Finally, Section 6 summarizes our work and concludes the paper.

## 2. Related Work

Query and integration of relational data in distributed systems can be realized in three paradigms: with central repository, with federation, and with peer-to-peer organization. In the central repository paradigm, structural or semantic data are crawled from data sources and put into a centralized data store. In the federation paradigm [4, 5], data are distributed. A centralized portal is used to receive all data requests, decompose them, and finally distribute the resulting requests to appropriate data stores. Peer-to-peer paradigm itself can be classified into two forms: unstructured and structured. Structured P2P systems build around the theory of distributed hash table which uses flat identifiers to store and locate data [6, 7]. Using flat identifiers, however, is not suitable for complex semantic queries. Unstructured P2P systems, on the other hand, have the potential to employ more complex semantics to query data. Early days' unstructured P2P systems, as well as sensor networks and mobile networks [8–10], rely on flooding or random walk to locate data, which has poor scalability. In response, several methods are proposed to address the scalability issue, for example, range query [11], multidimensions query [12], and the routing indices (RIs) [3]. RIs provide a list of “directions” towards the potential content sources for the query, which is similar to our semantic guider. However, the “directions” information maintained in a node's indices is a list of “coarser” topics and the number of files falling into each topic summarized from its nearby neighbors, which cannot fully support complex semantic queries (e.g., queries with relation constraints).

Support of semantic queries over large-scale networks has attracted much attention in recent years. In the layered semantic overlay networks (SONs) [13], nodes with semantically similar contents are “clustered” together, which makes network topology dependent on the contents and causes substantial maintenance overhead. In the SQPeer middleware [14] approach, each peer has to broadcast its data schema—which includes all RDF classes and properties—to (or requested by) other peer nodes to support semantic queries.

Most recently, several content centric networking approaches [15–19] are proposed to elevate content/information to the first-class citizen in the network. Data-Oriented (and beyond) Network Architecture (DONA) [15] focuses on content-based anycast. It uses flat, self-certifying names to identify content and relies on the Resolution Handlers (RHs) infrastructure to route a content request to the appropriate data source. Publish/Subscribe for Internet Routing Paradigm (PSIRP) [16] proposes a publish/subscribe based networking architecture. In Content Centric Network (CCN) [17], contents are identified by hierarchical names, and these names are announced, aggregated, and propagated into the routing system. It uses the guided information flooding model to route a request towards potential source(s). When a request is being forwarded, it leaves bread crumbs in the Pending Interest Table (PIT), which is later used to forward data packets back to the requester(s)(PIT is also used to suppress requests for the same content). In addition, CCN also supports transparent in-network content caching to reduce data traffic.

These aforementioned content centric networking approaches, however, mainly focus on facilitating users to efficiently retrieve a single named content. They do not tackle the problem of querying the network for relevant data with semantic constraints. The approach of similarity content search [20] moves one step further. Based on CCN, it introduces “search” as a top level namespace and uses flooding to search similar objects in a network, but it does not consider complex queries. Zahariadis et al. [21] have presented an Autonomic Layer-Less Object Architecture (ALLUA) framework which assigns different types of properties to content objects. From these properties, one can know several things such as the creator of the object, its relationship with other objects, and the way it is used.

In short, although there are various distributed index techniques and distributed query models, none of them can effectively deal with the semantic media data query and integration in large-scale networks. However, this capability is becoming increasingly important as network is more and more considered as a large distributed data store with rich semantic media than a simple communication medium.

## 3. Relation Routing Model

In this section, we introduce our relation routing model. We choose to extend the CCN to meet the goal of effectively dealing with the semantic media data query and integration in large-scale networks. Several reasons motivate the selection of CCN as our starting point. Firstly and most importantly, CCN's several design principles, such as name-based routing, guided information flooding model, soft-state PIT design, and in-network caching, naturally fit the needs of semantic query over large-scale networks. Secondly, CCN can be layered upon the existing Internet infrastructure or directly upon layer 2 networks, which has good potential of deployment. Finally, CCN is an open source which we can modify as we want.

Certainly, the goal of RRS is quite different from CCN. CCN focuses on the retrieval of a single named content, whereas RRS emphasizes on the ability to specify complex semantic queries and retrieve related data satisfying relation constraints. This distinction is the root cause of many differences between CCN and RRS, such as naming, packet format, forwarding procedures, and cached content.

### 3.1. Naming and Semantic Query Format

In rich media networking, since the semantic relation depends mainly on data itself, it is natural to use the name of data as well as its associated properties to identify and summarize its content. Because hierarchical namespace is very useful when one does not know the exact name of a data but has in mind only a data category [22] and hierarchical names are easy to be aggregated, we use the hierarchical namespace and adopt the URN in each level. However, different from traditional hierarchical names, where each part of the name corresponds to a location or organization related domain, our hierarchical names are solely based on semantic classification of data.

**Figure 1:**
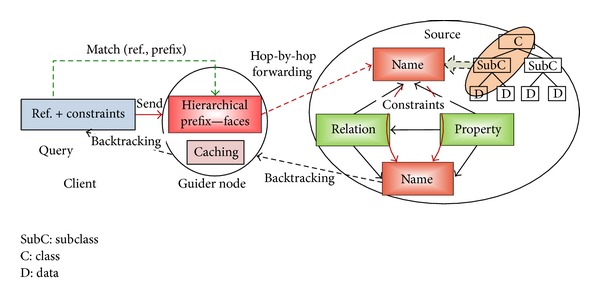
The interactions between “semantic guider” and other network entities.

We propose *semantic guider node* (SGN) that is able to give multigranularity directions towards the right sources for a referenced subject. With hierarchical namespace, every SGN only needs to hold the *hierarchical prefix* about the name of data rather than all the semantics of data to direct queries to right sources. The relationship between SGN and other network entities is briefly illustrated in Figure 1. A user's query can be represented by a reference subject (represented as a hierarchical name) plus constraints on the subject. The query will be forwarded by SGNs towards potential sources. When the query finally arrives at right sources, the constraints are used to extract matched data. The results will be returned and may be integrated on their way back.

In order for SGNs on different nodes to communicate and cooperate with each other, it is necessary that all SGNs conform to the same standard; that is, metadata of the name, relation, and property of data on all SGNs should be uniformly defined. Interaction between SGNs and heterogeneous data sources, however, requires transformation so that standard user queries are transformed to source-specific query languages and response results are transformed back to standard format metadata representation. In this way, heterogeneity of data sources is shielded from users.

Usually, a query can have property or relation constraints. We thus define two kinds of basic query formats: (1) query with property constraint; (2) query with relation constraint. The detailed formats of the queries are as follows.

1. “Prefix + property constraint”: the prefix is a hierarchical classification that restricts the subject of the query. The property constraint is a mixed logical and arithmetic function on any property of the given “prefix”, which is in the form:  〈*property*1 *MOP* *V*1 *LOP* *property*2 *MOP* *V*2…〉, where MOP is an arithmetic operator, *V*1 or *V*2 are predefined values, and *LOP* is a logical operator. For example, if a user wants to query the media of the subject (prefix) “*computer*/*network*”, with creation time (*T*) to be within the last 2 years and media format (*F*) to be “MPG”, then the constraint can be represented as “*p*{*T* > *Year*(“*NOW*”) − 2∧*F* = “*MPG”*}”.
2. “Prefix (+name) + relation constraint”: the prefix is a hierarchical classification or the hierarchical classification plus a “name” as the major reference subject. The relation constraint expresses the relation between the prefix (+name) and the data that the user wants. It is in the form of 〈*relation* 1  *OP* *relation* 2  *OP*…〉, where the *OP* is a logical operator. For example, if a user wants to query all the precursors and successors of the known media (or prefix) *b* constrained by relations *R* and *S*, the constraint can be represented as “*r*{*R*(∗, *b*)∧*S*(*b*, ∗)}”.

The two basic query formats can be used to describe various semantic queries in a unified format “hierarchical prefix + constraints”. To compactly express semantic queries, we adopt the relation algebra method. Nevertheless, such expressions can be easily rewritten in existing query languages (e.g., SQL or RQL [23]). In this paper, we focus on the second query format, as the routing processes of the two kinds of query formats are the same and the only difference lies in the procedures taken when the query reaches potential data sources.

### 3.2. Packet Structures

Two types of packets are defined in RRS: relation query packets and relation response packets. They are shown in Table 1.

1. *Relation Query Packet *(*Table 1*(*a*)). The packet contains four fields: Name, Selector, Scope, and Nonce. In the *Name* field, the user defines a referenced hierarchical name and/or prefix of the subject of interest, plus additional relation and/or property constraints. The *Selector* and *Scope* fields can be used to narrow the query's scope. In order to completely retrieve the relational data for a pending query, the SGN does not discard the pending query as soon as a response packet arrives. It waits some additional time to receive other related results from other sites. The time to wait is determined by the settings in the *Scope* field. While a user is querying for media content, he/she can estimate and set the value of certain query parameters, such as estimated maximum round trip time, publisher, and so forth. These items are defined in the *Selector* field. The *Nonce* field is inherited from CCN's request packet, used to avoid packet forwarding loops.
2. *Relation Response Packet *(*Table 1*(*b*)). The packet is mainly used for carrying a list of relational data with additional information, which also contains four fields: Name, Publisher ID, Validation Key, and Data. The *Name* field is simply copied from the counterpart in the relation query packet. The *Data* field carries relational data that matches the query. The *Publisher ID* field identifies the publisher of the relational data. And the *Validation Key* field contains information needed to verify the validity of the relational data, for example, public key.

### 3.3. Forwarding Engine of SGN

**Figure 2:**
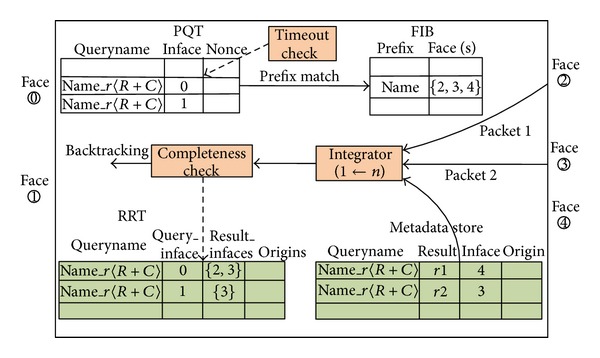
The forwarding and integration engine for relation routing scheme.

In RRS, the main task of SGN is to offer query guidance, data backtracking, and result integration. We extend the forwarding engine of CCN to support the relation routing and result integration, which is shown in Figure 2. Besides the Pending Interest Table (PIT) (renamed as Pending Query Table in RRS model for reasons as below), Forwarding Information Base (FIB), and content store(renamed as metadata store for reasons as below), a new data structure called Response Relation Table (RRT) is added to the engine. The following is a detailed description of these components.

1. *Pending Query Table *(*PQT*). Similar to the PIT in CCN, PQT is used to temporarily record the semantic queries that have been forwarded but still wait for responses. PQT also follows the soft-state design principle, which means that each entry will be deleted after a reasonable period of time. However, the choice of when to delete the query in PQT is different from CCN. In CCN, a request is for a definitely named content, so the relationship between request and response is 1 : 1. Hence, the query in PIT can be deleted immediately after one copy of the data returns, whereas in RRS, the relationship between semantic query and response is 1 : *n*; that is, a semantic query may result in many different results, so it is not appropriate to delete the entry immediately after one response returns.
2. *Forwarding Information Base *(*FIB*). FIB stores the names and/or the prefixes of all media that are distributed across the whole network and the outgoing faces(Here face refers to the port that is used to exchange information directly with their neighbors for application processes) through which they can be reached them. Except for the semantic distinctions in names, the FIB in RRT is the same as in CCN.
3. *Response Relation Table *(*RRT*). RRT is a newly introduced data structure, which is mainly used to handle the incomplete response problem introduced in Section 3.4. Briefly speaking, the incomplete response problem also arises from the 1 : *n* relationship between semantic query and response. In case when different users issue the same query, it is possible that some users can only get incomplete responses. RRT is used to record the presently returned results for a query, so that incomplete response to a query can be detected.
4. *Metadata store*. Unlike CCN's content cache, which caches the data content itself, the metadata store in RRS caches relation response results and related information (such as publisher). This information gives detailed descriptions of the data contents, including the means to fetch the contents. Despite this distinction, the purpose of metadata store is similar to the content store in CCN, that is, to satisfy popular queries from cache, rather than forwarding the query outward each time. Traditional cache replacement policies, such as *Least Recently Used* (LRU), can be used.

The major attributes as well as their descriptions for these components (except for FIB) are given in Table 2.

### 3.4. The Incomplete Response Problem

Since the data of interest to a user may be distributed over several data sources, it is important to retrieve the response packets from multiple sources. However, when different users issue the same query (i.e., the name and preference are the same), it is possible that some users only get incomplete responses (or no response packets at all) before the due time of the pending query. For example, consider the situation when user A has sent out a query *Q*1 and got *partial (or complete)* response packets, and before the deletion time of its PQT record, some user B sends out the same query *Q*1. This query will be suppressed and combined in the PQT. If the metadata store evicts the cached results of *Q*1 before B's query arrives, then early responses to *Q*1 before the arriving time of B's query will be lost. This is called the *incomplete response* problem. To address this issue, we take two modifications to the basic CCN forwarding engine.

Firstly, when a first response packet is received, the SGN does not remove its query record in PQT immediately(in CCN, the query is removed immediately after the expected content arrives); instead, it sets a due time according to two options. One is the timeout option, which determines how much time the pending query takes to be deleted after it has arrived. Generally speaking, the due time is affected by several factors such as the network transit delays, the duplicate number of the referenced subject, and the complexity of the query. Another option is the “maximum number of response packets” option, which determines how many packets to be received before the pending query can be deleted.

**Figure 3:**
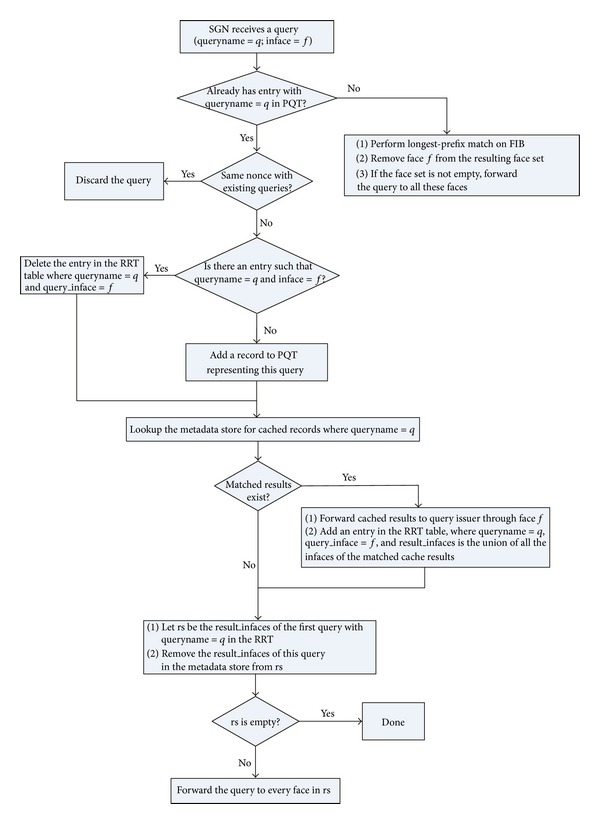
Query process logic.

Secondly, we introduce an additional data structure, called *Response Relation Table* (RRT). This table is used to temporarily record which query results have been returned to corresponding pending queries in PQT. The major fields of the RRT are shown in Figure 2. When an SGN receives a query, it will take different actions depending on whether queries with the same query name have been received within a given time interval. If the query is the first one with this query name, then the SGN will search the FIB with the longest prefix match algorithm and forward the query to all matching faces (except the arriving face). Otherwise, if the query is not the first one, then the SGN will first look up the metadata store and return any results that match the query. Because it is possible that early arrived results can be evicted by a cache replacement policy, it is required to validate the cache completeness. The basic solution is to assume that the Response Relation Table for the first query with this query name records all the incoming faces of the results that have so far been returned, so by comparing this incoming faces with the incoming faces of the results for the query in the metadata store, it is possible to deduce whether the cached result is complete. If the cached result is incomplete, that it is necessary to forward the query to *some faces (the difference between the above two incoming faces)* to ensure response completeness. The complete flow chart of the query processing logic of SGN is shown in Figure 3.

Certainly, it is necessary to periodically check whether records in PQT are timeout (note that only one timer is set for all pending queries with the same queryname), which is not shown in the flow chart. For those pending queries, that all response packets have been sent back before timeout, the associated RRT records are also deleted. Otherwise, the query should be reforwarded to appropriate faces.

## 4. Relation Integration

When multiple response packets are available, it is necessary to integrate these results and filter redundant ones before forwarding the results back so that computation and bandwidth overhead can be reduced. This is what relation integration does. A relation integrator accepts multiple inputs and produces one output by logical integration.

In the following, we first introduce several relation operations and four basic integrations and then show how complex semantic query processing can be facilitated through these basic operations.

### 4.1. Relation Operations

Relation operations can be executed on SGNs or the data sources where a set of media related to a given query can be integrated. It can also be executed on the client node before the results are presented to the user. Four basic operations are defined.

- *Ranking*. This operation is used to sort the elements in multiple relation response packets based on property or relationship. For example, regarding the query “*C*1/*C*2/*C*3_*r*{⋯}”, if an SGN has received 3 result packets, {*e*1} for prefix “*C*1/*C*2/*C*3”, {*e*2} for prefix “*C*1”, and {*e*3} for prefix “*C*1/*C*2”, the results can be sorted as *e*1 > *e*3 > *e*2 if ranked by the length of matched prefix.
- *Intersection*. This operation is used to select common data out of multiple relation response packets. For example, {*b*1, *b*2}∪{*b*2, *b*3} = {*b*2}.
- *Union*. This operation is used to merge all relational data packets of the same query and remove duplicated ones. For example, two relation response packets are merged: {*b*1, *b*2}∪{*b*2, *b*3} = {*b*1, *b*2, *b*3}.
- *Complement*. This operation is used to select differential data from multiple relation response packets. For example, {*b*1, *b*2}∖{*b*2, *b*3} = {*b*1, *b*3}.

### 4.2. Basic Relation Integration

Four basic relation integrations are defined: forward composition, backward composition, joint composition, and bridging composition. These basic integrations can be combined to produce more complex integrations. In the following, we first introduce the four basic integrations and then show how to use these basic integrations to generate more complex ones.

**Figure 4:**
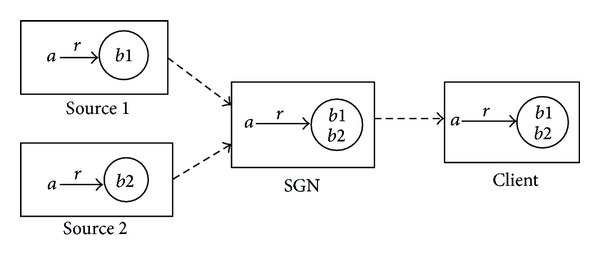
The integration process of the forward composition.

**Figure 5:**
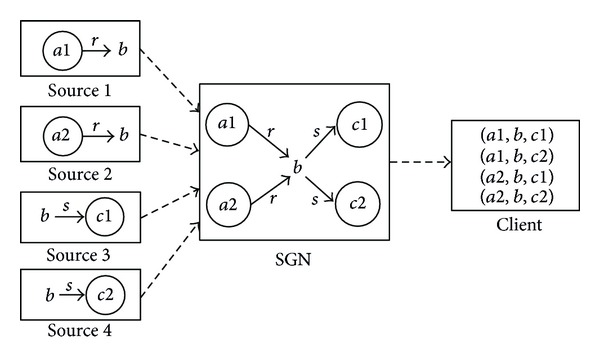
The integration process of the joint composition.

**Figure 6:**
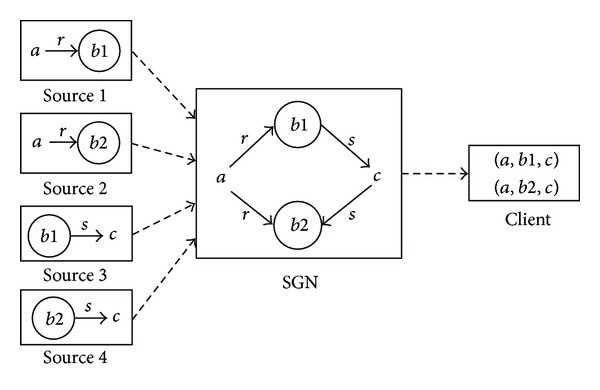
The integration process of the bridging composition.

(1) *Forward Composition*. The composition can be expressed as *R* | (*a*, ∗) = {(*a*, *b*)*R*(*a*, *b*)}, and the name of the query can be defined as “*a*_*r*〈*R*(*a*, ?)〉”. It is used to retrieve and integrate the media objects that are the successors of the prefix (+name) *a*, and the relationship between the data object *a* and these media data is *R*.  Figure 4 gives an example of forward composition. When the query is executed at two potential sources, source 1 and source 2, the subresults *r*(*a*, *b*1), *r*(*a*, *b*2) will be returned and can be combined at either the SGN or the client.

(2) *Backward Composition*. The backward composition is opposite to the forward composition, which can be expressed as *R* | (∗, *b*) = {(*a*, *b*) | *R*(*a*, *b*)}. The name of the query can be represented as “*b*_*r*  〈*R*(?, *b*)〉”. The integration process of the backward composition is similar to that of the forward composition.

(3) *Joint Composition *(*Jcomp*). This composition can be represented as *R*∗*S* | (∗, *b*, ∗) = {(*a*, *b*, *c*)*R*(*a*, *b*)∧*S*(*b*, *c*)}, and the name of the query can be represented as “*b*_*r*  〈*Jcomp*(*R*(?, *b*), *S*(*b*, ?))〉”. It is used to integrate all the precursors and successors of the known media (or prefix) *b* with relation constraints *R* and *S*.  Figure 5 visually exemplifies this integration process. When the query is executed at the potential sources, suppose that the subresults are *R*(*a*1, *b*) in source 1, *R*(*a*2, *b*) in source 2, *S*(*b*, *c*1) in source 3, and *S*(*b*, *c*2) in source 4 and can be returned to the guider node. The joint composition *b*_*r*  〈*Jcomp*(*R*(?, *b*), *S*(*b*, ?))〉 at the guider node will result in four data sequences, that is, (*a*1, *b*, *c*1), (*a*1, *b*, *c*2), (*a*2, *b*, *c*1), and (*a*2, *b*, *c*2).

(4) *Bridging Composition* (*Bcomp*). This composition can be represented as *R*∗*S* | (*a*, ∗, *c*) = {(*a*, *b*, *c*) | *R*(*a*, *b*)∧*S*(*b*, *c*)}. The name of the query can be represented as “*a*∘*c*_*r*  〈*Bcomp*(*R*(*a*, ?), *S*(?, *c*))〉”. It is used to integrate the media data that are associated with a known precursor and a known successor with relation constraints *R* and *S*. The integration process of the bridging composition is illustrated in Figure 6. When the query is executed at the potential sources, and the subresults are *R*(*a*, *b*1) at source 1, *R*(*a*, *b*2) at source 2, *S*(*b*1, *c*) at source 3, and *S*(*b*2, *c*) at source 4, the bridging composition “*a*∘*c*_*r*  〈*Bcomp*(*R*(*a*, ?), *S*(?, *c*))〉” at the guider node will result in two data sequences, that is, (*a*, *b*1, *c*) and (*a*, *b*2, *c*).

### 4.3. More Complex Integrations

The above basic compositions can be used to generate a variety of complex integrations of practical use. In this subsection, we illustrate four complex integrations.

(1) *Iterative Forward Composition *(*IFC*). It iteratively uses the forward composition to generate longer data sequences consecutively constrained by the same relationship *R*. The integration can be defined as *R* | (*a*, ∗∗) = {(*a*, *b*, *c*,…) | *R*(*a*, *b*)∧*R*(*b*, *c*)∧⋯}. The name of its query is expressed as “*a*_*r*  〈while  *R*(*a*, ?) + *constraint*〉”, where *constraint* describes the integration restriction, such as the length limit of generated sequences. For example, if a user wants to generate the sequences that start with object *a*, consecutively constrained by relationship *R*, and the length is no greater than 3, the query name can be expressed as “*a*_*r*〈*while* *R*(*a*, ?) + “ ≤ 3*”*〉”.

(2) *Iterative Backward Composition *(*IBC*). Similar to IFC, IBC iteratively uses backward composition to generate longer data sequences consecutively constrained by the same relationship *R*. This integration can be defined as *R* | (∗∗, *a*) = {(…, *c*, *b*, *a*) | *R*(*b*, *a*)∧*R*(*c*, *b*)∧⋯}. The name of its query is represented as “*a*_*r*  〈while  *R*(?, *a*) + *constraint*〉”, where *constraint* is the same as that of IFC.

(3) *Base Hybrid Composition *(*BHC*). BHC is to generate longer data sequences that link specified objects and expected objects constrained by different relationships. In this integration strategy, a user can specify several objects as well as the relationships between the specified objects and the expected objects. For example, given the objects *a*, *c*, and *e*, the relationships *R*1, *R*2, *R*3, *R*4, and *R*5, the subsequence among the objects *a* and *c* is the bridging composition with relationships *R*1 and *R*2, the sub-sequence near the object  *e*  is the joint composition with relationships *R*4 and *R*5,  and the relationship between the object *c* and the precursor of the object *e* is *R*3. So this integration can be expressed  as  *R*1∗*R*2∗*R*3∗*R*4∗*R*5 | (*a*, %, *c*, %, *e*, %)= {(*a*, *b*, *c*, *d*, *e*, *f*) | *R*1∗*R*2 | (*a*, ∗, *c*), *R*4∗*R*5 | (∗, *e*, ∗), *R*3(*c*, *d*)}, and the name of  its query can be represented  as “*a*_*r* 〈*Bcomp*(*R*1(*a*, ?), *R*2(?, *c*)), *Jcomp*(*d* = *R*4(?, *e*), *R*5(*e*, ?)), *R*3(*c*, *d*)〉”. 

(4) *Limited Hybrid Composition *(*LHC*). The integration strategy can generate long sequences with various relationships and length constraints. According to the referenced object from which a sequence is generated, LHC can be categorized into three kinds: (1) limited hybrid composition starting from the front (LHC-F); (2) limited hybrid composition starting from the middle (LHC-M); (3) limited hybrid composition starting from the end (LHC-E). Here, we elaborate on the first kind. For example, consider composing a finite sequence which starts from object *a*. The subsequence (*a*, *b*, *c*) is constrained by relationship *R*1, subsequence (*c*, *d*, *e*) is constrained by relationship *R*2, and subsequence (*e*, *f*) is constrained by relationship *R*3. The integration strategy can be represented as *R*1(2)*R*2(2)*R*3(1) | *a* = {(*a*, *b*, *c*, *d*, *e*, *f*) | *R*1(2)∣(*a*, ∗∗), *R*2(2)∣(*c*, ∗∗), *R*3 | (*e*, ∗)}. The value in parenthesis “( )” behind the relationship describes the length limit of the subsequence. The name of this query can be written as “*a*_*r* 〈(*b*, *c*) = while  *R*1(*a*, ?) + “ ≤ 2”, (*d*, *e*) = while  *R*2(*c*, ?) + “ ≤ 2”,  *f* = *R*3(*e*, ?)〉 ” or *“a*_*r* 〈*b* = *R*1(*a*, ?),  *c* = *R*1(*b*, ?),  *d* = *R*2(*c*, ?),  *e* = *R*2(*d*, ?), *f* = *R*3(*e*, ?)〉”.

## 5. Performance Evaluation

We evaluate the system performance of RRS from both analytical and experimental perspectives. 

### 5.1. Analytical Evaluation

In this subsection, we analytically evaluate the performance of RRS from several aspects: index precision and completeness, routing scalability, and reliability.

#### 5.1.1. Index Precision and Completeness

Index precision means the extent to which a user's query can be accurately routed to potential sources. Since the FIB in RRS is built in the same way as in CCN, which holds media names or prefixes advertised by all media sources, the precision and completeness of indices of RRS should be the same as in CCN. In comparison, since RIs only maintain local indices, their precision and completeness should be lower than RRS.

#### 5.1.2. Routing Scalability

The routing protocols used in RRS and CCN are similar to conventional routing protocols in the present-day Internet, such as BGP, whose scalability have already been demonstrated. The main difference lies in naming. The hierarchical naming mechanism can describe different classes of media and be used to perform matching at different granularities, which greatly improves the ability to cope with the diversity of user queries. Prefix aggregation can be used to condense the FIB size (e.g., “computer/network/TCP” and “computer/network/UDP” into “computer/network”), provided that the aggregation will not result in routing uncertainty. Other software or hardware enhancements could also be adopted to improve the forwarding and matching efficiency, such as those being developed in the ongoing NDN (Named Data Networking) project [24].

#### 5.1.3. Reliability

**Figure 7:**
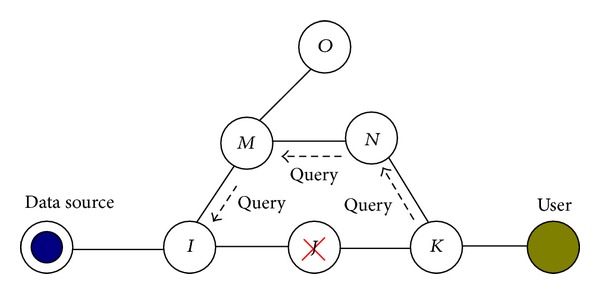
Solution for recovery of the return path.

If a node in the network goes offline, the query accessibility (query accessibility means reachability between the query source and potential data sources) should be maintained and the backtracking path for the results should be resumed. Query accessibility is an important factor that affects the results returned. Since the name is independent of location, a mechanism similar to the Opaque LSA [25] in the OSPF protocol can be added to guider nodes to describe the state of its neighbors and update the corresponding faces in FIB and PQT when the neighbor changes. If a guider node can propagate all prefixes to its neighbors in due time, queries can be guided to potential sources. In order to resume a broken backtracking path due to node or link failure, it needs to discover a redundant return path for response packets. One possible solution is illustrated in Figure 7. When a node (say *J*) fails, its adjacent neighbors (*I*, *M*, and *K*) can sense this failure and then readvertise their routing table to their neighbors (according to the routing protocol). After the routing table stabilizes, the downstream node *K* of the failed node then resends the query toward the data source. When the query reaches the upstream node *I* of the failed node, *I* replaces the invalid face of *J* with the new face of *M*, hence, recovers the return path. In addition, reliability is also benefited from the increase in duplication, either by source or by cache.

### 5.2. Comparative Evaluation

Table 3 summarizes the important features of the RRS and some of the current distributed media query schemes. In Table 3, we compare the following aspects that dominate the query performance of these systems.

(1) *Query Description*. Most systems consider either complex user requirements without taking into account the routing or simply defined requirement for routing. We have considered both requirement description (constraints) and routing information (prefix) included in the query of RRS.

(2) *Query Model*. Before the advent of the P2P networks, most systems should provide a global view or central index structure for the purpose of distributed query. P2P networks do not have this requirement, but efficiently delivering complex user requests to the right sources cannot be handled well. To deal with all these issues, we employ the prefix-based routing in the RRS in view of the prefix provided by the user query. 

(3) *Dynamic Adaptability*. It is not easy for traditional systems to retrieve the data distributed across a network when the data moves from one place to another before the system updates the global view or indices. The RRS has the advantage of dynamic adaptability even when the name and position of all distributed data are changing, which is similar to the unstructured P2P network. 

(4) *Transmission Optimality*. Most systems build the query system above the communication facilities of network, so they cannot identify the local duplicates of data on the network. The RRS, however, can quickly access the local content and support transparent in-network content caching to cut short the transmission path. 

(5) *Acquisition Directivity*. Most systems have considered the query direction provided by global view, central indices, or distributed hash table except for unstructured P2P networks. However, all those schemata should be limited to the global view, central indices, or distributed hash mapping to show the right direction towards certain host(s). To the RRS, the direction is easily established by all sources multicasting all the prefixes of content names. 

(6) *Search Capability.* Generally, we consider the query capability from the recall and precision of search. In this way, the distributed database has a stronger capability for searching the data among the federated database systems by structural query language (e.g., SQL). But the search capability of the unstructured P2P network relies on the semantic matching capability of forwarding nodes as it imposes no constraint on the query format and the web search engine cannot provide the high precision of search due to the keywords search pattern. However, the RRS can provide much stronger search capability by using unified names on the network and carrying semantic constraints in the query.

### 5.3. Experimental Evaluation

#### 5.3.1. Experiment Setup

**Figure 8:**
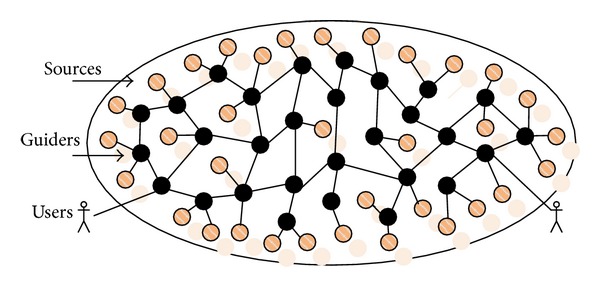
The schematic topology of the experimental network (sources are more than guider nodes).

**Figure 9:**
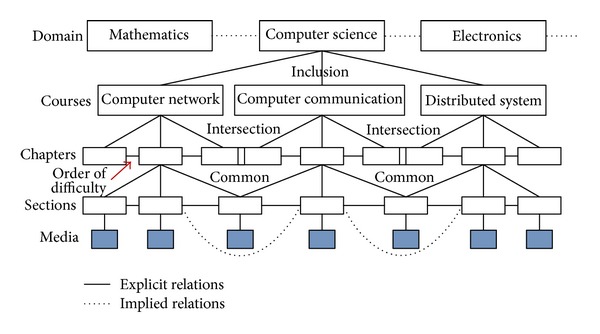
The relations of partial media data.

To evaluate the functionality and efficacy of RRS, we build an overlay network with 2074 nodes on a PC cluster composed of 68 physical computers. We randomly select 1054 nodes as data sources and take the others as guider nodes. Data nodes are randomly connected to one or more SGNs. The CPU of these computers are Intel Pentium 4, and the RAMs range from 512 MB to 1 GB. Each data source stores relational media data about all or part of 30 college courses, and the relational data (more than 3 thousand records at each source) are stored in MySQL databases. The semantic description of local relational media at all sources conforms to a uniform name and relation specification. We set the number of duplicates (duplicate factor) of individual media objects (name or prefix) to be between 1 and 16. These duplicates are distributed over different sources. A conservative threshold of 3 minutes is used for the residence time of each arrived query in the PQT. The schematic topology of the experimental network is shown in Figure 8. Relations of partial media data are shown in Figure 9, which gives the complex relations, such as hierarchical classification, inclusion, and intersection, between course media data. In addition, we assume that users are directly connected to guider nodes rather than to data sources.

In the following, we evaluate the efficiency and cost of our relation routing model with different number of duplicates and different number of hops between the user and the data sources.

#### 5.3.2. Experimental Results

**Figure 10:**
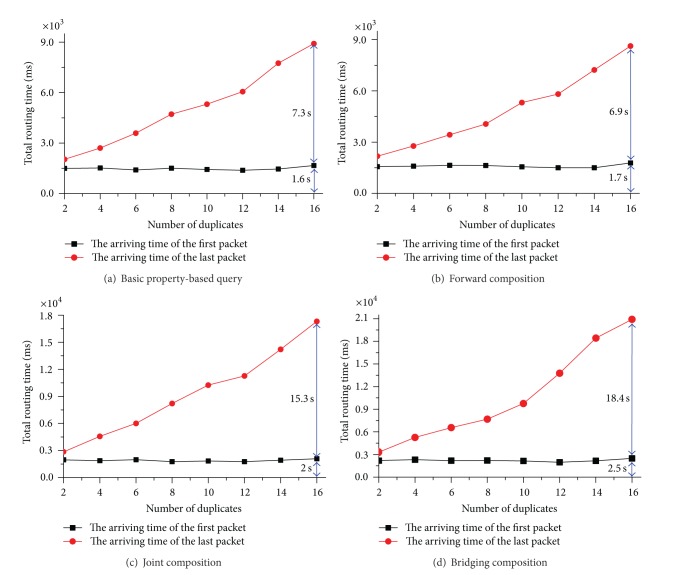
Testing results for total routing time with different duplicates (hops are 6).

We first look at the routing efficiency of different queries with various duplication factors.  Figure 10 shows the total routing time of the queries with property and relation constraints. The query with relation constraints include forward composition, joint composition and bridging composition. The total routing time is the time from sending out an query until the reception of the last response packet.

In the first experiment, we fix the average number of hops from client node to data sources to be 6 and vary the duplication factor from 2 to 16. We repeat each query more than 10 times and average the results. Figures 10(a)–10(d) present the arrival time of the first and last response packets for four queries (basic property based query, forward composition (we omit backward composition here because it is similar to forward composition), joint composition, and bridging composition), respectively. The arriving time of the first response packet shows that it can retrieve the metadata from closest sources in a very short interval. And the arriving time of the first response keeps being stable as the duplication factor increases. The arriving time of last response packets shows that the total routing time increases slowly as the duplication factor increases. The four kinds of media queries can all be completed in a few seconds even when the duplication factor is 16.

This is not surprising because the sources are distributed, the query and responses are concurrently transmitted over the network, and the number of transmission paths from the user to the duplicates of reference subject is increasing as the duplication increases.

**Figure 11:**
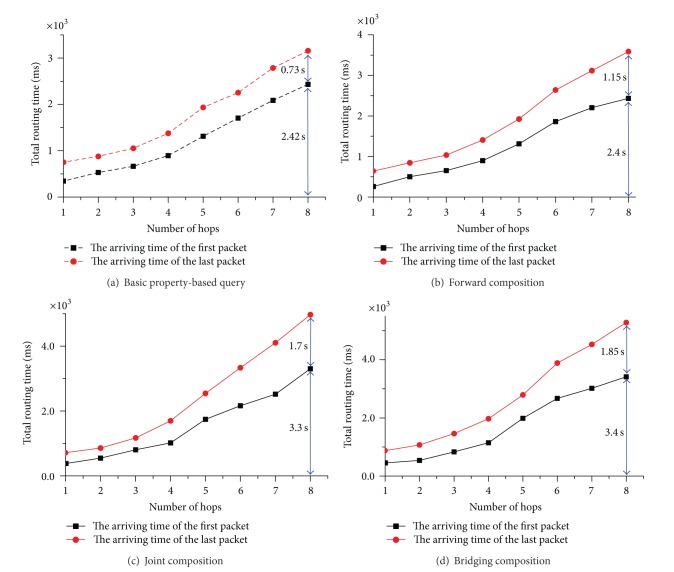
Testing results for total routing time with different hops (duplicates are 3).

In the second experiment, we fix the duplication factor to be 3 but vary the number of hops from client to data sources from 1 to 8.  Figure 11 shows the total routing time for the four kinds of queries mentioned earlier. The results indicate that the arriving times of the first and last response packets both increase slowly as the number of hops increases.

This implies that the complete, decentralized results can be returned to user in a short time.

It should be pointed out that the total query processing time is short for each integration query because the size of relational data, the forwarding time, and the average number of routing hops are all very small in real networks.

**Figure 12:**
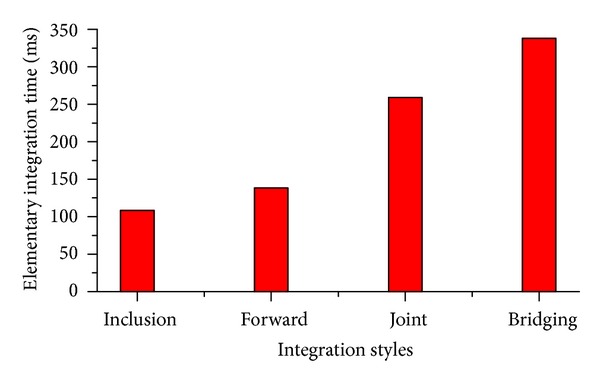
Testing results for elementary integration time.

Figure 12 presents the elementary integration time of the 4 types of queries. The elementary integration time refers to the average time that is spent for querying data and performing an elementary integration at each data source. The results indicate that the elementary integration time is less than 1 s for these basic integration styles. Finally, the results reveal that the computation complexities are very low and it is larger for the bridging composition than others.

## 6. Summary

With the development of media technologies, efficient support of complex semantic media queries over large-scale networks will greatly enhance the sharing of large decentralized media and provide a powerful data integration service for users.

For this purpose, we proposed a *relation routing scheme* (RRS) and described its makeups. The semantic-guider is the core component in RRS. By extending the CCN forwarding engine to implement the semantic guider, we inherit many merits from CCN and hope it will have good opportunity to be incrementally deployed. However, RRS and CCN have very different purposes, which makes them differ in several ways. First, RRS adopts a semantic based hierarchy naming convention rather than location or organization based hierarchy naming convention because RRS mainly deals with semantic query. Second, RRS's query format should support complex property and relationship constraints. Thirdly, the 1:*n* relationship between query and responses in RRS results in different timeout strategy of the pending query, the introduction of a new component called RRT to handle incomplete response problem, and the relation integration to condense relevant results and filter redundant ones. Finally, the cache no longer caches the content itself but the relational metadata about the content. Both analytical analysis and experimental studies based on a prototype implementation demonstrated the feasibility and efficiency of this new scheme.

Certainly, it should be acknowledged that there are many possible optimizations that can be made to this scheme, for example, optimization of RRS for high scalability and resilience. Also, the performance evaluation should be carried out to justify the appropriateness of various designing choices and parameter settings, such as the correlation between the query complexity and response time. These are all in our future research agenda.

## Figures and Tables

**Table:** (a) Relation query packet

Name	Query name in the form of name_r<constraints>
Selector	Order preference, publisher filter
Scope	Response quantity, timeout, and so forth
Nonce	Time stamp or sequence number to avoid routing loops

**Table:** (b) Relation response packet

Name	Same as in the relation query packet
Publisher ID	Identification of the publisher for this relational data
Validation Key	Information needed to verify the validity of relational data.
Data	A sequence of relational data results

**Table 2:** Major attribute names and their descriptions for the PQT, RRT, and metadata store.

Attribute name	Description
Pending Query Table	
Queryname	Name of the query represented in the format as discussed in Section 3.1
Inface	The incoming face of the query
Nonce	The nonce of the query packet for loop avoidance

Metadata store	
Queryname	Same as in PQT
Result	Result of the query
Inface	Incoming face of the result
Origin	Source that generates the result

Response Relation Table	
Queryname	Same as in PQT
Query_inface	Incoming face of the query
Result_infaces	Incoming faces of the results that have already been forwarded through query_inface for this query
Origns	Sources that generate these results

**Table 3:** Comparison of distributed media query schemes.

Scheme	Distributed database	Structural P2P network	Unstructured P2P network	Web search engine	RRS
Query description	Structural query language (e.g. SQL)	Set(s) of flat identifiers (e.g. DHT)	No limited the forms	Keywords plus Boolean operations	Prefix + semantic constraints
Query model	To query global view	To match structural mapping between identifier and location	Flooding, random walk, supernode routing, and so forth	To query the centrical indexes	Prefix-based routing
Adaptable to dynamic sources	Poor	Good	Very good	Poor	Very good
The optimality of transmission path	Poor	Poor	Poor	Poor	Very good
Acquisition directivity	Good	Very good	Poor	Very good	Very good
Search capability	Stronger	Weak	Rely on the matching capability of forwarding nodes	Normal	Stronger
